# Two Doses of BNT162b2 mRNA Vaccine in Patients after Hematopoietic Stem Cell Transplantation: Humoral Response and Serological Conversion Predictors

**DOI:** 10.3390/cancers14020325

**Published:** 2022-01-10

**Authors:** Maciej Majcherek, Agnieszka Matkowska-Kocjan, Donata Szymczak, Magdalena Karasek, Agnieszka Szeremet, Aleksandra Kiraga, Aneta Milanowska, Edwin Kuznik, Krzysztof Kujawa, Tomasz Wrobel, Leszek Szenborn, Anna Czyz

**Affiliations:** 1 Department of Hematology and Bone Marrow Transplantation, Wroclaw Medical University, 50-367 Wroclaw, Poland; maciej.majcherek@umw.edu.pl (M.M.); donata.szymczak@umed.wroc.pl (D.S.); agnieszka.szeremet@umw.edu.pl (A.S.); tomasz.wrobel@umw.edu.pl (T.W.); 2 Department of Pediatric Infectious Diseases, Wroclaw Medical University, 50-368 Wroclaw, Poland; agnieszka.matkowska.kocjan@interia.pl (A.M.-K.); Leszek.szenborn@umw.edu.pl (L.S.); 3 University Hospital in Wroclaw, 50-556 Wroclaw, Poland; magdalena.karasek@student.umed.wroc.pl; 4 Laboratory of Flow Cytometry and Cytomorphology, Department of Hematology and Bone Marrow Transplantation, University Hospital in Wroclaw, 50-556 Wroclaw, Poland; akiraga@usk.wroc.pl (A.K.); amilanowska@usk.wroc.pl (A.M.); 5 Department of Angiology, Hypertension and Diabetology, Wroclaw Medical University, 50-529 Wroclaw, Poland; edvin@op.pl; 6 Statistical Analysis Centre, Wroclaw Medical University, 50-368 Wroclaw, Poland; Krzysztof.kujawa@umw.edu.pl

**Keywords:** anti-SARS-CoV-2 vaccination, hematopoietic stem cell transplantation, hematological malignancies, efficacy, humoral response, immune response predictors

## Abstract

**Simple Summary:**

Vaccination against SARS-CoV-2 is currently the best tool in the fight against the COVID-19 pandemic. However, there are concerns about its efficacy and safety after hematopoietic stem cell transplantation. The aim of the study was to study the efficacy and safety of two doses of BNT162b2 mRNA vaccine in adult patients after autologous or allogeneic transplantation. We examined the presence of anti-SARS-CoV-2 antibodies before and after vaccination. We also searched for the potential predictors of serological conversion after vaccination, including the analysis of the impact of various lymphocyte subpopulations at the time of vaccination on post-vaccine antibody concentration and seroconversion. In addition, patients were followed-up for adverse events after vaccination, and the data on breakthrough SARS-CoV-2 infection were collected. The results of our study broaden the knowledge of the efficacy and safety of BNT162b2 mRNA vaccine in patients after HCT, providing new data on serological conversion predictors.

**Abstract:**

Vaccination against SARS-CoV-2 is currently the best tool in the fight against the COVID-19 pandemic. However, there are limited data on its efficacy and safety after hematopoietic stem cell transplantation (HCT). We present the results of a prospective analysis of the humoral response to two doses of BNT162b2 mRNA vaccine in 93 adult patients, including 29 after autologous HCT (autoHCT) and 64 after allogeneic HCT (alloHCT). Positive anti-SARS-CoV-2 antibodies were detected before vaccination in 25% of patients despite a negative medical history of COVID-19. Seroconversion after vaccination was achieved in 89% of patients after alloHCT and in 96% after autoHCT, without grade 3/4 adverse events. Post-vaccination anti-SARS-CoV-2 antibody level correlated with the time from transplant and absolute B-cell count at the vaccination. In univariate analysis restricted to the alloHCT group, short time since transplantation, low B-cell count, low intensity conditioning, GvHD, and immunosuppressive treatment at the vaccination were associated with lack of seroconversion. In the multivariate model, the only negative predictor of seroconversion remained treatment with calcineurin inhibitor (CNI). In conclusion, the BNT162b2 mRNA vaccine is highly immunogenic in patients after HCT, but treatment with CNI at the time of vaccination has a strong negative impact on the humoral response

## 1. Introduction

According to a WHO report, over 270 million cases of SARS-CoV-2 infection have been registered worldwide, and the COVID-19 epidemic has claimed over 5 million lives [1]. Apart from fatalities, COVID-19 carries many other serious complications, especially in hematological patients [2]. Due to the low effectiveness of available drugs against COVID-19 [3], the best tool in the fight against this disease is the strategy of wide-spread vaccination. The necessity of carrying it out in a wide group of people with various clinical conditions causes many questions about the effectiveness and safety of vaccinations [4].

A particular group is patients with hematological malignancies. As shown by many studies in this population, SARS-CoV-2 infection is associated with a significantly higher risk of progression to the severe form of COVID-19 and a worse prognosis if this occurs [5,6].

The other risk is also a poorer response to vaccination in patients with hematological malignancies compared to the general population (74.6% vs. 99.1%) [7]. However, the risks associated with vaccination appear to be far less important than the potential benefits, even if the vaccine is only partially effective [8]. In addition, it is also worth noting that the effectiveness of vaccinations in patients with hematological malignancies also depends on the type of cancer or the vaccine used [9].

Therefore, many scientific societies, including the Polish Society of Hematology and Transfusion Medicine, have issued recommendations for vaccination against SARS-CoV-2 in patients undergoing the transplant procedure as soon as three months after the procedure [10].

In a population of solid-organ transplant recipients requiring chronic immunosuppression, BNT162b2 mRNA vaccination was significantly less effective than in a healthy population [11]. Available literature data reveals that patients after allogeneic hematopoietic stem cell transplantation (alloHCT) or after chimeric antigen receptor T lymphocyte (CAR-T) treatment who are not on immunosuppression respond well to vaccination with high levels of anti-SARS-CoV-2 IgG [12].

Not only has the effectiveness of the COVID vaccine been in question, but safety in these patients is also a subject of concern. Fortunately, the data published so far suggest that mRNA vaccines are safe in patients with blood cancers, even after the third dose [13].

The most important remaining questions associated with vaccination of transplant patients are: the possibility of increased cytopenia after the use of recombinant mRNA vaccines, the risk of occurrence or the possible severity of graft versus host disease (GvHD), and the graft versus tumor (GvT) phenomenon influence. All these doubts need to be resolved to ensure that patients are offered a safe form of vaccination.

The aim of our study was to analyze the humoral response to two doses of BNT162b2 mRNA vaccine in adult hematological patients after autologous or allogeneic HCT. We also searched for potential predictors of seroconversion after vaccination, including the impact of clinical features and different lymphocyte subpopulations at the time of vaccination on post-vaccine antibody concentration and seroconversion. In addition, we assessed the safety of BNT162b2 mRNA vaccine in this particular patient population.

## 2. Materials and Methods

### 2.1. Study Design

The study was designed to include 100 patients after autologous or allogeneic hematopoietic stem cell transplantation. Patients transplanted in the Department of Hematology, Blood Neoplasm and Bone Marrow Transplantation of Wroclaw Medical University qualified for vaccination in accordance with the guidelines of the National COVID-19 Immunization Program valid in March 2021 and the recommendations of EBMT [10]. The study group consisted of patients after autologous transplant performed in 2020 and patients after alloHCT, regardless of the year when the procedure was performed; however, at least 3 months after HCT according to the recommendations. The immune status before and after vaccination, as well as the efficacy and safety of the BNT162b2 mRNA vaccine, were prospectively assessed.

The protocol was conducted according to the Declaration of Helsinki, approved by the local ethics committee, and informed consent was obtained from all patients.

The clinical data and transplantation details, including the date of transplantation, donor type, conditioning regimen, GvHD prophylaxis and the time of its discontinuation, and the occurrence of acute or chronic GvHD and its treatment, were obtained from institutional medical records. The disease risk index (DRI) [14] and toxicity conditioning intensity (TCI) [15] score were assessed retrospectively.

Vaccinations were performed by administering two doses of BNT162b2 mRNA vaccine 5 weeks apart (the shortest available interval at this time—due to vaccination shortages in March/April 2021 the Polish government decided to extend the interval between the vaccine doses from the initially recommended 3 weeks). Preparation of vaccine doses and administration followed the manufacturer’s guidelines. Vaccinations were performed by trained and experienced medical staff at the Medical University of Wroclaw vaccination center in March/April 2021. Due to safety reasons (SARS-CoV-2 infection threat), the study patients were separated from the rest of the vaccinated population.

Blood samples were collected twice. The first collection was initially scheduled within 14 days before vaccination, and the second sampling two to four weeks after vaccination [16]. However, due to patients’ decisions and/or logistical considerations reported by the patients, the second blood sample collection in some patients were performed later, five to six weeks after vaccination. At both time points, the level of antibodies against SARS-CoV-2, peripheral blood leukocyte and lymphocyte subpopulations, and the overall serum concentrations of immunoglobulins IgA, IgG, and IgM were determined. Samples were collected by qualified nursing personnel from the cephalic vein.

### 2.2. Flow Cytometry

Peripheral blood tests were performed on an eight-color BD FACSCanto II cytometer (Becton Dickinson and Company (BD), San Jose, CA, USA). Staining was performed according to standard facility procedures, with the panel of antibodies presented in Table 1. The antibodies were added in the amounts recommended by the manufacturer to 200 μL of whole blood. After 15 min of incubation with antibodies, lysis of erythrocytes was performed with BD FACS Lysing Solution, followed by two wash steps. Acquisition was performed within an hour after staining (average 4 × 10^5^ events). The test was performed in a single tube. Data were analyzed in the BD FACS Diva 8.0.1 Software (Becton Dickinson and Company, BD Bioscience, San Jose, CA, USA). The dual-platform method was used to determine absolute cell counts.

### 2.3. Method of Anti-SARS-CoV-2 Antibody Evaluation

Anti-SARS-CoV-2 antibody levels were measured with chemiluminescent microparticle immunoassay (CMIA) “Alinity I” from Abbott Diagnostics—a two-step qualitative and semi-quantitative assay designed to detect IgG antibodies to the receptor binding domain of the S1 subunit of the spike protein of SARS-CoV-2 in serum or plasma.

### 2.4. Total Immunoglobulin Concentation Evaluation

Serum concentrations of immunoglobulins IgA, IgG, and IgM were assessed by a commercial nephelometry assay.

### 2.5. Vaccination Safety Assessment

A week after each dose of the vaccine, all patients were asked about the state of their health and the possible adverse events they had experienced after vaccination according to a unified protocol. The patients’ last follow-up for breakthrough SARS-CoV-2 infection and survival was updated on 5 November 2021.

### 2.6. Statistical Analysis

Descriptive statistics and nonparametric tests were applied to compare groups, either the Mann–Whitney rank test, where appropriate, or the Wilcoxon matched pairs signed-rank test. Nonparametric Pearson chi-squared and Fisher’s exact tests were used to evaluate differences across patients with and without seroconversion after vaccination. In descriptive summaries, the modified version of the geometric mean was applied to the sets of numbers with zero values, in which zero values (0) were converted to one (1) for the calculation.

The correlations between anti-SARS-CoV-2 antibody levels and ordinal or continuous variables (including age, time since HCT to vaccination, the transplant conditioning intensity score, baseline counts and percentages of lymphocyte subpopulations, and baseline concentration of immunoglobulin G, M, and A) were analyzed using Spearman’s correlation coefficient.

Predictive effects of selected baseline clinical characteristics and laboratory markers for anti-SARS-CoV-2 IgG antibody response to vaccination were assessed by univariate and multivariate logistic regression. Predictors that were found to be related to seroconversion (*p* ≤ 0.10) in univariate analysis were then entered into a multivariable logistic regression model using stepwise forward selection. If two or more predictors were strongly correlated, only one was used when building the multivariable logistic model. It enabled avoiding collinearity of the predictors, in line with the multivariable regression model’s assumption. *p* < 0.05 was considered statistically significant. Statistical analyses were performed with the STATISTICA version 13.3 package (STATSOFT Polska, Kraków, Poland).

## 3. Results

### 3.1. Patients and HCT Procedures

A total of 93 patients were included in the study. The patients’ most important baseline characteristics and transplant details are presented in Table 2. The majority of study patients (69%) underwent allogeneic HCT. The main indication for alloHCT was acute myeloid leukemia (55% of patients). Among patients after autologous HCT (autoHCT), 48% were treated due to multiple myeloma, and the remaining patients were transplanted for lymphoma. The median time between allogeneic HCT and vaccination was 23 (range 3–112) months, while that between autologous HCT and vaccination was 10 (range 4–38) months. Peripheral blood was the graft source in both autologous and allogeneic HCT. The intensity of the conditioning regimens in the alloHCT group was determined according to the Transplant Conditioning Intensity (TCI) score [15] as low, intermediate, or high. For GvHD prophylaxis, antithymocyte globulin was administered to all but one patient transplanted from a matched unrelated donor, and posttransplant cyclophosphamide was administered to all patients undergoing haploidentical HCT and a patient transplanted from a 9/10 matched unrelated donor. At the time of vaccination, 18 of 64 patients (28%) after alloHCT were receiving immunosuppressive treatment, including 10 patients who were treated with calcineurin inhibitors (CNIs). In the autoHCT group, one patient with mantle cell lymphoma was treated with rituximab and ibrutinib as posttransplant maintenance therapy.

### 3.2. The Presence of Anti-SARS-CoV-2 Antibodies at Baseline Prevaccination Testing

All study patients had no documented medical history of COVID-19 prior to vaccination. At baseline, the presence of anti-SARS-CoV-2 IgG antibodies was assessed in all patients. A total of 23 patients (25%) were anti-SARS-CoV-2 IgG positive, including 5 of 29 after autoHCT (17%) and 18 of 64 (28%) after alloHCT.

### 3.3. Anti-SARS-CoV-2 Antibodies at Post-Vaccination Testing

Anti-SARS-CoV-2 antibodies after vaccination were assessed in 89 out of 93 patients, including 26 after autoHCT and 63 after alloHCT. Postvaccination antibody testing was not performed in four patients in this group due to patient decisions or organizational reasons. Seroconversion was achieved in 56 of 63 patients (89%) in the alloHCT group and in all but one patient (96%) in the autoHCT group.

The postvaccination geometric mean concentration (GMC) of anti-SARS-CoV-2 antibodies was 1157.73 BAU/mL with a median concentration of 3292.24 (range, 0.00–56,729.00) BAU/mL. In the subgroup of patients who achieved seroconversion, GMC and the median concentration of antibodies were 2317.16 BAU/mL and 3736.13 (range, 23.02–56,729.00) BAU/mL, respectively.

As 22 patients returned for a second blood test after the originally scheduled date of the second sampling, i.e., more than 4 weeks after vaccination, we performed an additional comparison of antibody levels in 67 patients who had a scheduled test, i.e., from 2 to 4 weeks after vaccination, with levels in 22 patients who were tested later, 5 to 6 weeks after vaccination. No difference was found between these two groups (GMC 859.21 vs. 1809.09 BAU/mL, the median concentration 3573.15 vs. 3207.98 BAU/mL; *p* = 0.85). Additionally, GMC and the median concentration of antibodies were compared between patients who had a second sampling up to 39 days after vaccination (*n* = 79), with those tested 40 to 42 days after the second dose (*n* = 10), and similarly, no significant difference was found (GMC 1001.11 vs. 1448.46 BAU/mL, the median concentration 3673.81 vs. 2052.06 BAU/mL; *p* = 0.26).

### 3.4. Analysis of Humoral Response Predictors

The first attempt was to correlate post-vaccination antibody concentration with baseline clinical and laboratory characteristics, such as the patient age, time between HCT and vaccination, absolute lymphocyte count, percentage, and absolute count of B lymphocytes, CD4+ and CD8+ T lymphocytes, CD3+CD4− CD8− lymphocytes, NK cells, and monocytes. We found a positive correlation with the time between HCT and vaccination (r = 0.44, *p* < 0.0001). Moreover, the antibody level also showed a moderate positive correlation with the B-cell count at vaccination (r = 0.42, *p* < 0.0001) and the percentage of B lymphocytes (r = 0.54, *p* < 0.0001). In addition, a weak positive correlation with the CD4+ T-cell count (r = 0.27, *p* = 0.011) was found.

The comparison of post-vaccination antibody concentrations according to baseline patient characteristics is shown in Table 3. Significantly higher antibody levels were seen in women, in patients who were vaccinated after a longer time after transplantation, and in those with pre-vaccination positive anti-SARS-CoV-2 antibody concentrations. In the alloHCT group, the antibody level was higher in patients without GvHD symptoms at the time of vaccination and in those without immunosuppressive treatment. When patients were categorized according to ongoing treatment with calcineurin inhibitor (CNI) at the time of vaccination, the difference in antibody concentration was also highly significant.

### 3.5. Analysis of Post-Vaccination Serological Conversion

In the autoHCT group, only one patient did not achieve seroconversion. The patient underwent autoHCT due to mantle cell lymphoma and was on maintenance therapy with rituximab and ibrutinib at the time of vaccination, as the only patient in the autoHCT group. In the alloHCT group, 56 of 63 patients (89%) achieved seroconversion. Six of the seven seronegative patients after vaccination were treated with calcineurin inhibitor (CNI) at the time of vaccination, including one additionally receiving prednisone and one mycophenolate mofetil. Two of these six patients on CNI were treated for acute GvHD, two for chronic GvHD, and two others were still on GvHD prophylaxis after alloHCT. One remaining seronegative patient was treated topically for chronic ocular GvHD.

The comparison of patients with and without seroconversion after vaccination is shown in Table 4. The absolute count and percentage of various lymphocyte subpopulations in these two groups are shown in Table 5. The time interval between HCT and vaccination was shorter in patients without seroconversion (Table 4). In addition, patients without seroconversion had lower baseline B-lymphocyte counts and B-lymphocyte percentages in blood and tended to be older (Table 5). When the alloHCT group was analyzed separately, patients without seroconversion were more likely to receive immunosuppressive treatment at the time of vaccination and were conditioned with less intensive regimens according to the TCI score (Table 4).

The logistic regression analysis of seroconversion predictors was restricted to the alloHCT group. The results of univariate and multivariate analyses are presented in Table 6. The B-lymphocyte count, patient age, and time interval between alloHCT and vaccination were introduced in the model as continuous variables, TCI score as an ordinal variable, and CNI treatment as a categorical variable. As the two predictors, i.e., the B-lymphocyte count and CNI treatment, were strongly correlated (Z = 2.07, *p* = 0.036), only CNI treatment was used when building the multivariable logistic model. Similarly, because GvHD at the time of vaccination was strongly correlated with CNI treatment (*p* < 0.001), GvHD was not introduced in the model. Finally, in multivariate analysis, the only factor that remained an independent negative predictor of antibody response was treatment with CNI at the time of vaccination.

### 3.6. Changes in Complete Blood Count and Differential Leukocyte Count after Vaccination

No changes were found in complete blood count or differential leukocyte count after vaccination, as presented in Table 7. The only change noticed after vaccination was an increase in absolute lymphocyte and absolute monocyte counts. In addition, we also noticed a significant increase in overall IgM and IgA levels after vaccination. The median serum concentrations of IgM before and after vaccination were 0.52 g/dl (range, 0–2.55) and 0.66 g/dl (range, 0–4.31), respectively (*p* = 0.0099). The respective serum concentrations of immunoglobulin A were 0.96 g/dl (range, 0–4.90) and 1.04 g/dl (range, 0–7.62) (*p* = 0.001). In contrast, we did not find an increase of IgG levels after vaccination (9.18 g/dl vs. 8.90 g/dl; *p* = 0.26).

### 3.7. Post-Vaccination Graft-Versus-Host Disease Clinical Course

At the time of vaccination, 12 patients were treated for GvHD, including four with acute GvHD and eight with chronic GvHD. After vaccination in six patients, no change in the GvHD clinical course was observed; in four patients, improvement was noticed, and two patients experienced deterioration of GvHD symptoms. No new-onset GvHD was observed after vaccination, and no impact of vaccination on the GvHD rate or severity was found.

### 3.8. Adverse Events

The patients complained only about mild adverse effects typical of vaccination with the BNT162b2 mRNA COVID-19 vaccine (pain at the injection site, headache, fatigue, chills). We did not observe any grade 3 or higher adverse events according to the Common Terminology Criteria for Adverse Events ver. 5.0 (CTCAE) [17].

### 3.9. Breakthrough SARS-CoV-2 Infection

All patients were alive at the last follow-up in November 2021. Only one patient in the study group, transplanted from MUD in 2018, developed asymptomatic SARS-CoV-2 infections six months after the second dose of vaccine.

## 4. Discussion

Although vaccination seems to be the most effective method of fighting the COVID-19 pandemic, its use is associated with many concerns that need to be addressed, including safety and effectiveness in vulnerable populations such as transplant patients. To enhance the published experience, we examined the anti-SARS-CoV-2 antibody concentration before and after two doses of BNT162b2 mRNA vaccine in this particular group of patients. An interesting phenomenon observed in our study group was the presence of anti-SARS-CoV-2 antibodies prior to vaccination despite a negative medical history of COVID-19. This indicates that many of the patients had undergone COVID-19 asymptomatically or trivially, as previously reported. Shah found that up to 35% of transplant patients had experienced asymptomatic or mild COVID-19 [18]. Importantly, individuals with positive baseline serology showed significantly higher antibody levels after vaccination than patients with basically negative antibody test results. In addition, we revealed higher antibody concentrations after vaccination in women, which is in line with the observations in the general population [19]. However, due to the relatively small number of patients in our study group, this result should be interpreted with caution. We also found a stronger immunologic response in younger patients, which is an expected result in concordance with observations of COVID-19 mRNA vaccine immunity in the general population [20,21]. Interestingly, we also found an association between transplant conditioning intensity in alloHCT and seroconversion after vaccination.

The seroconversion rate was higher in patients after alloHCT with intermediate or high TCI than in those with low TCI. Historically, conditioning regimens prior to allogeneic HCT have been classified as myeloablative, reduced intensity, and non-myeloablative. However, due to the difficulties of classifying many of the new regimens, the new conditioning toxicity intensity score was introduced in 2019 [15]. This allows the comparison of transplants in which various conditioning regimens are used. To our knowledge, the efficacy of posttransplant vaccinations in groups of patients who received different conditioning regimens in terms of TCI score has not been compared yet. Moreover, we also presented higher antibody concentrations in patients in later periods after transplantation, and we revealed an association between the time from transplant to vaccination and postvaccination seroconversion, which is in line with other authors’ observations [22,23].

Among the factors affecting the development of antibodies against SARS-CoV-2 after vaccination in HCT recipients, the total lymphocyte number, the number of B and NK lymphocytes, as well as the administration of rituximab, have been reported so far [24,25]. According to our data, both the number and percentage of B lymphocytes correlate positively with the antibody concentration and with seroconversion after vaccination. In our study group, only one patient received rituximab at vaccination as maintenance therapy after autologous HCT, and this patient was the only patient in the autologous transplant group who did not develop antibodies. This finding seems to be confirmed by data from studies evaluating seroconversion in patients treated with rituximab [26]. Although we did not show a correlation between NK lymphocyte count and antibody level, as previously reported [24], we observed a weak positive correlation with CD4+ lymphocytes. However, neither CD4+ lymphocyte count nor percentage was confirmed as a predictor of humoral response in logistic regression analysis.

The vast majority of patients after allogeneic HCT developed antibodies after vaccination completion. However, in subjects treated with immunosuppressants at the time of vaccination, the response rate and antibody levels were significantly lower than in subjects without immunosuppressants. This is like the observations made in solid-organ transplantation (SOT) recipients (who routinely require chronic immunosuppressive treatment), in whom the immunologic response rate was only 17–28% after two doses of the vaccine [27,28]. In general, in HCT recipients, the use of immunosuppressants is a factor associated with an unfavorable response to vaccination [29]. We also confirmed the negative impact of the use of calcineurin inhibitors on humoral response after vaccination, which remained the only independent predictor of seroconversion after vaccination in multivariate logistic regression. Interestingly, this observation was not confirmed in the studies carried out by a Spanish group. Their publication showed that the use of steroids in high doses is a stronger predictor than the use of CNI. The discrepancy may be explained by the fact that in our group there were no patients treated with high doses of steroids. This confirms the necessity of further searching for predictors in a wide group of patients after alloHCT [25]. In such clinical settings, a reasonable approach seems to be the administration of an additional third dose of the vaccine, which increases the percentage of patients with positive anti-SARS-CoV-2 antibodies [30,31]. In August/September 2021, for HCT patients who were still receiving immunosuppressive treatment or who were less than 2 years after the HCT procedure, the third (additional) dose of the COVID-19 vaccine was recommended in many countries [32].

Currently, the effectiveness of vaccinations is mainly assessed by the presence of antibodies [12]. Nevertheless, full evaluation of vaccine benefit requires further studies, including assessment of T-cell responses [33] and the “real world” efficacy against SARS-CoV-2 infection or severe COVID-19. Of note, due to the reports of SARS-CoV-2 breakthrough infections in vaccinated patients, clinical observations will be most important [34]. Moreover, it should be noted that so far no protective level of antibodies was determined [35].

It is worth mentioning that only one patient in our study group developed asymptomatic SARS-CoV-2 infection during the follow-up time of 6 months after vaccination. Our observations may suggest high vaccination efficacy in both auto- and allotransplant recipients and are consistent with the findings of Spanish researchers who after vaccination revealed only one recipient of alloHCT (0.2%) with breakthrough SARS-CoV-2 infection [25]. However, further studies are needed to assess the clinical efficacy of vaccinations in various epidemic conditions and over a longer period of observation.

In accordance with our data, no severe post-vaccination reactions were found in patients after hematopoietic stem cell transplantation, which corresponds to other studies on this subject. Due to the reports in the literature of the occurrence of immune thrombocytopenia and hemolytic anemia after vaccination against SARS-CoV-2, concerns have arisen as to whether vaccination in hematological patients after HCT will cause an increase in rates and severity of cytopenia [36,37]. From our observations, no statistically significant changes in platelets and absolute neutrophil count or hemoglobin concentration were found. We observed only individual patients whose blood counts declined, which was interpreted as related to the progression of the underlying hematological disease rather than to vaccination itself.

In the group of allogeneic HCT recipients, a specific posttransplant complication, graft-versus-host disease, needs to be addressed. Since a dysregulation of immune mechanisms is responsible for the pathogenesis of this condition [38], the safety of COVID vaccination in transplant patients has been called into question. In our experience, vaccinations did not affect the occurrence and clinical course of GvHD. Another issue is the impact of GvHD on the efficacy of vaccination. As stated in previous publications, the effectiveness of various vaccines in patients with GvHD may be lower [39,40]. In our study group, individuals with GvHD developed lower concentrations of anti-SARS-CoV-2 antibodies. However, it should be emphasized that all but one was on systemic immunosuppressive agents.

Due to difficulties with vaccine supply, the patients included in the study received vaccinations according to a schedule other than that recommended by the manufacturer and used in other studies. However, humoral response rates were similar to those reported in other publications [24,25]. This indicates that two doses of the vaccine are effective, even when the vaccination interval is extended.

Our study group differed in terms of diagnosis, prior treatment, type, and time from HCT. As a result, the populations in the respective groups were relatively small. However, despite this, it was possible to find factors influencing vaccination efficacy, also in a multivariate analysis.

## 5. Conclusions

According to our data, the BNT162b2 mRNA vaccine is safe and highly immunogenic in patients after HCT, ensuring seroconversion in the vast majority of patients after both autologous and allogeneic transplant. Vaccination is less effective in alloHCT recipients who receive immunosuppressants in the course of GvHD, especially in those treated with calcineurin inhibitors. Observations are required to evaluate vaccination real-world efficacy in preventing COVID-19.

## Figures and Tables

**Table 1:** Panel of antibodies used for flow cytometry.

Antigen	CD3	CD4	CD8	CD14	CD19	CD16/CD56	CD45	HLA-DR	CD20
Fluorochrome	APC	PerCP-Cy5.5	FITC	APC-H7	PC7	PE	V500	V450	PerCP-Cy5.5
Clone	SK7	SK3	SK1	MφP9	J3-119	B73.1/MY31	2D1	L243	2H7
Producer	BD	BD	BD	BD	BC	BD/BD	BD	BD	BD

BD, Becton Dickinson and Company, San Jose, CA, USA; BC, Beckman Coulter, Inc., Brea, CA, USA.

**Table 2:** Baseline patients’ characteristics at SARS-CoV-2 vaccination.

Characteristic	autoHCT	alloHCT
Number of patients	29	64
Median (range) age in years	58 (26–69)	52 (20–68)
Patient gender		
Female	13 (45%)	30 (48%)
Male	16 (55%)	34 (52%)
Diagnosis		
AML	0	36 (55%)
MDS	0	7 (11%)
ALL	0	5 (9%)
NHL	11 (38%)	6 (9%)
HL	4 (14%)	3 (5%)
MM	14 (48%)	0
Other	0	7 (11%)
Median (range) time between HCT and vaccination in months	10 (4–38)	23 (3–112)
Donor type		
HLA-identical sibling	NA	7 (11%)
HLA-matched unrelated (9/10 or 10/10)	NA	52 (81.5%)
Haploidentical	NA	5 (7.5%)
Stem cell source		
Peripheral blood	29 (100%)	64 (100%)
ATG in GvHD prophylaxis		
Yes	NA	51 (80%)
No	NA	13 (20%)
Post-transplant Cy		
Yes	NA	6 (10%)
No	NA	58 (90%)
Progressive disease at HCT		
Yes	0	0
No	29(100%)	64 (100%)
Patient on IS at vaccination		
Yes	NA	18 23%
No	NA	46 (77%)

HCT, hematopoietic stem cell transplantation; auto, autologous; allo, allogeneic; IS, immunosuppression; AML, acute myeloid leukemia; MDS, myelodysplastic syndrome; ALL, acute lymphoblastic leukemia; MM, multiple myeloma; HL, Hodgkin lymphoma; NHL, non-Hodgkin lymphoma; ATG, antithymocyte globulin; Cy, cyclophosphamide; NA, not applicable.

**Table 3:** Humoral response after SARS-CoV-2 vaccination by baseline patients’ characteristics.

Characteristics	All Patients				Patients with Seroconversion			
	*N*	Anti-SARS-CoV-2 (Anti-S Protein) Antibody Concentration after Vaccination		*p*	*N*	Anti-SARS-CoV-2 (Anti-S Protein) Antibody Concentration after Vaccination		*p*
		GMC	Median (Range)			GMC	Median (Range)	
Age								
≤60 years	57	1738.58	3818.85 (0–18,599.00)	0.079	54	2631.52	3843.92 (23.02–18,599.00)	0.24
>60 years	32	561.12	1476.43 (0–56,729.00)		27	1796.62	2236.19 (33.20–56,729.00)	
Gender								
Female	42	1787.52	3859.54 (0–56,729.00)	0.05	39	3017.57	3951.14 (43.14–56,729.00)	0.05
Male	47	785.29	2536.46 (0–14,115.00)		42	1701.15	2932.93 (23.02–14,115.00)	
Anti-SARS-CoV-2 antibodies before vaccination								
Positive *	22	6628.27	7340.50 (394.76–56,729.00)	<0.001	22	6628.27	7340.50 (394.76–56,729.00)	<0.001
Negative **	67	549.73	2472.15 (0.00–18,599.00)		59	1565.87	2900.82 (23.02–18,599.00)	
Type of HCT								
AlloHCT	63	919.97	3123.73 (0–56,729.00)	0.82	56	2063.64	3741.02 (23.02–56,729.00)	0.82
AutoHCT	26	2020.74	3677.95 (0–24,160.00)		25	2739.88	3682.09 (43.14–24,160.00)	
Time between HCT and vaccination								
≤12 months	39	366.89	1190.39 (0–24,160.00)	<0.001	33	1066.10	2236.19 (23.02–24,160.00)	<0.001
>12 months	50	2837.14	4066.18 (0–56,729.00)		48	3951.40	4197.57 (252.42–56,729.00)	
Transplant Conditioning Intensity score in alloHCT group								
Low	20	180.11	947.90 (0–56,729.00)	0.16	14	1599.67	2194.96 (33.20–56,729.00)	0.16
Intermediate/ high	39	1822.67	3858.99 (0.7–18,599.00)		38	2241.78	3908.55 (23.02–18,599.00)	
Missing data	4				4			
GvHD at vaccination in alloHCT group								
No	51	2064.80	3858.99 (0–56,729)	<0.001	49	2739.05	3958.12 (71.62–56,729.00)	<0.001
Yes	12	29.62	28.11 (0–5393.84)		7	383.90	328.38 (23.02–5393.84)	
Post-alloHCT IS treatment at vaccination								
Yes	18	57.24	76.79 (0–5393.84)	<0.001	12	424.90	529.31 (23.02–5393.84)	<0.001
No	45	2793.79	3991.25 (0–56,729.00)		44	3345.92	40167.2 (90.50–56,729.00)	
Post-alloHCT treatment with calcineurin inhibitor at vaccination								
Yes	10	8.79	2.05 (0–4314.63)	<0.001	4	216.52	84.37 (71.62–4314.63)	<0.001
No	53	2212.24	3818.85 (0–56,729.00)		52	2401.63	3779.93 (23.02–56,729.00)	

IS, immunosuppressive; HCT, hematopoietic stem cell transplantation; alloHCT, allogeneic HCT, autoHCT, autologous HCT; GMC, geometric mean concentration; *N*, number * ≥ 7.1 BAU/mL, ** < 7.1 BAU/mL.

**Table 4:** Patients’ baseline characteristics at SARS-CoV-2 vaccination by serological conversion after vaccination.

Characteristics	*N*	All Patients		*p*	*N*	Patients after alloHCT		
		Patients with Seroconversion *N*	Patients without Seroconversion *N*			Patients with Seroconversion *N*	Patients without Seroconversion *N*	*p*
	89	81	8	NA	63	56	7	
Median (range) age in years		53 (20–69)	63 (33–68)	0.1		49 (20–69)	63 (33–68)	0.1
Gender								
Female	42	39 (48%)	3 (37.5%)	0.72	30	27 (48%)	3 (43%)	0.80
Male	47	42 (52%)	5 (62.5%)		33	29 (52%)	4 (57%)	
Median (range) time since HCT to the vaccination in months		14.3 (3.3–111.9)	7.8 (3.3–15.8)	0.02		27.3 (4.9–111.8)	7.7 (3.3–15.8)	0.02
Type of transplant								
AutoHCT	26	25 (31%)	1 (12.5%)	0.43		NA	NA	
AlloHCT	63	56 (69%)	7 (87.5%)					
Transplant conditioning intensity (TCI) in the alloHCT group								
Low		NA	NA		20	14 (27%)	6 (86%)	0.005
Intermediate/high					39	38 (73%)	1 (14%)	
missing					4			
Active acute or chronic GvHD at vaccination								
Yes		NA	NA		12	7 (12.5%)	5 (29%)	<0.001
No					51	49 (87.5%)	2 (71%)	
Patients on post-alloHCT IS treatment at vaccination								
Yes		NA	NA		18	12 (21%)	6 (86%)	0.002
No					45	44 (79%)	1 (14%)	
Patients on post-alloHCT treatment with CNI at vaccination								
Yes		NA	NA		10	4 (7%)	6 (86%)	<0.001
No					53	52 (93%)	1 (14%)	

HCT, hematopoietic stem cell transplantation; alloHCT, allogeneic hematopoietic stem cell transplantation; IS, immunosuppressive; TCI, transplant conditioning intensity; CNI, calcineurin inhibitor; NA, not applicable.

**Table 5:** Lymphocyte subpopulations in peripheral blood at SARS-CoV-2 vaccination by serological conversion after vaccination.

Lymphocyte Subpopulations	All Pts		*p*	Pts after alloHCT		
	Pts with Seroconversion *N*	Pts without Seroconversion *N*		Pts with Seroconversion *N*	Pts without Seroconversion *N*	*p*
	77	7		53	6	
Median (range) of lymphocyte count in blood (G/L)	1.36 (0.33–6.67)	1.13 (0.79–3.77)	0.69	1.38 (0.33–6.67)	1.32 (0.79–3.77)	0.89
Median (range) of B-cell count in blood (G/L)	0.21 (0.00–1.11)	0.01 (0.00–0.35)	0.014	0.22 (0.0–1.11)	0.07 (0.0–0.35)	0.038
Median (range) percentage of B cells in blood (%)	13.5 (0.1–41.0)	1.5 (0.0–10.0)	<0.001	14.5 (1.40–41.0)	4.50 (0.04–10.00)	<.001
Median (range) of T-cell count in blood (G/L)	0.89 (0.23–5.86)	1.08 (0.42–2.90)	0.66	0.87 (0.23–5.86)	1.03 (0.42–2.89)	0.83
Median (range) percentage of T cells in blood (%)	66.9 (23.0–95.9)	76.8 (53.3–95.9)	0.098	65.9 (23.0–95.9)	23.95 (13.90–38.50)	0.27
Median (range) of CD4+ T-cell count in blood (G/L)	0.23 (0.04–0.73)	0.21 (0.1–0.64)	0.92	0.22 (0.04–0.73)	0.21 (0.09–0.64)	0.64
Median (range) percentage of CD4+ T cells in blood (%)	28.0 (9.4–70.1)	25.8 (13.9–38.5)	0.76	28.0 (9.4–70.1)	23.95 (13.9–38.5)	0.57
Median (range) of CD8+ T-cell count in blood (G/L)	0.61 (0.11–5.34)	0.64 (0.24–2.21)	0.80	0.57 (0.11–5.34)	0.64 (0.24–2.21)	0.72
Median (range) percentage of CD8+ T cells in blood (%)	70.4 (27.9- 91.1)	64.1 (56.8–82.3)	0.66	70.3 (27.9–91.1)	70.2 (56.8–82.3)	0.98
Median (range) of NK-cell count in blood (G/L)	0.13 (0.02–0.71)	0.15 (0.04–0.37)	0.50	0.12 (0.02–0.71)	0.17 (0.12–0.37)	0.10
Median (range) percentage of NK cells in blood (%)	15.2 (2.1–54.3)	12.8 (3.9–45.8)	0.99	13.9 (2.1–54.3)	18.7 (8.1–45.8)	0.47

Pts, patients; HCT, hematopoietic stem cell transplantation; alloHCT, allogeneic hematopoietic stem cell transplantation; IS, immunosuppressive; TCI, Transplant Conditioning Intensity; CNI, calcineurin inhibitor; NA, not applicable.

**Table 6:** Univariate and multivariate logistic regression analysis of factors associated with seroconversion after SARS-CoV-2 vaccination in patients after allogeneic hematopoietic stem cell transplantation.

Predictor	Univariate		Multivariate	
	Unadjusted OR (95% CI)	*p*	Adjusted OR (95% CI)	*p*
Age	0.92 (0.85–1.01)	ns		ns
Time since transplantation to vaccination	1.27 (1.01–1.59)	0.042		ns
B-cell count at vaccination	1.01 (1.00–1.01)	0.09		ns
GvHD at the vaccination	0.25 (0.11–0.39)	0.0004		ns
CNI treatment at the vaccination	0.01 (0.00–0.13)	0.0003	0.03 (0.00–0.79)	0.03
Conditioning intensity according to TCI score	16.29 (1.80–147.55)	0.013		ns

OR, odds ratio; CI, confidence interval; CNI, calcineurin inhibitor; TCI, transplant conditioning intensity; ns, not significant.

**Table 7:** Complete blood count and white-blood-cell count with differential before and after SARS-CoV-2 vaccination.

Laboratory Parameters	CBC and WBC with Differential before Vaccination *n* = 82	CBC and WBC with Differential after Vaccination *n* = 82	*p*
Mean ± SD of WBC (G/L)	5.02 ± 1.78	5.14 ± 1.85	0.10
Mean ± SD of absolute neutrophil count (G/L)	3.04 ± 1.23	2.92 ± 1.37	0.31
Mean ± SD of absolute lymphocyte count (G/L)	1.60 ± 0.96	1.79 ± 1.17	<0.001
Mean ± SD of absolute monocyte count (G/L)	0.38 ± 0.14	0.43 ± 0.17	<0.001
Mean ± SD of platelet count (G/L)	163 ± 67	165 ± 76	0.52
Mean ± SD of Hb concentration (g/dl)	14.1 ± 8.6	14.3 ± 13.4	0.11

CBC, complete blood count; WBC, white-blood-cell count; SD, standard deviation.

## Data Availability

The data presented in this study are available on request from the corresponding author. The data are not publicly available due to the protection of personal data.
